# PSBF: *p-adic* Integer Scalable Bloom Filter

**DOI:** 10.3390/s23187775

**Published:** 2023-09-09

**Authors:** Wenlong Yi, Chuang Wang, Qiliang Xie, Yingding Zhao, Jing Jia

**Affiliations:** School of Software, Jiangxi Agricultural University, Nanchang 330045, China; yiwenlong@jxau.edu.cn (W.Y.); wangchuang@stu.jxau.edu.cn (C.W.); xieqiliangg@outlook.com (Q.X.); zhaoyingding@jxau.edu.cn (Y.Z.)

**Keywords:** *p-adic*, number theory, access optimization, bloom filter, scalable

## Abstract

Given the challenges associated with the dynamic expansion of the conventional bloom filter’s capacity, the prevalence of false positives, and the subpar access performance, this study employs the algebraic and topological characteristics of *p-adic* integers to introduce an innovative approach for dynamically expanding the *p-adic* Integer Scalable Bloom Filter (PSBF). The proposed method involves converting the target element into an integer using a string hash function, followed by the conversion of said integer into a *p-adic* integer through algebraic properties. This process automatically establishes the topological tree access structure of the PSBF. The experiment involved a comparison of access performance among the standard bloom filter, dynamic bloom filter, and scalable bloom filter. The findings indicate that the PSBF offers advantages such as avoidance of a linear storage structure, enhanced efficiency in element insertion and query, improved storage space utilization, and reduced likelihood of false positives. Consequently, the PSBF presents a novel approach to the dynamic extensibility of bloom filters.

## 1. Introduction

**Figure 1:**
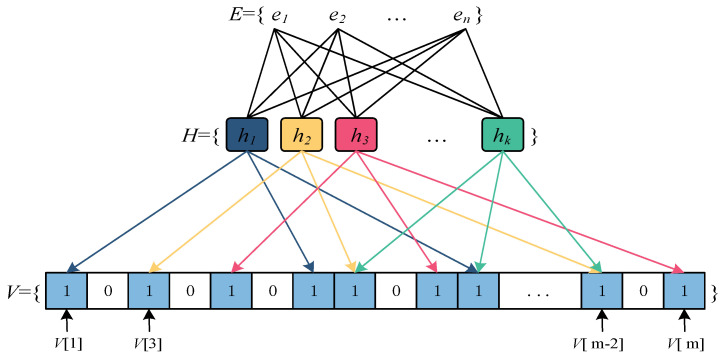
Data structure of a standard bloom filter.

The advent of contemporary information technologies, including big data, the Internet of Things, and cloud computing, has resulted in a substantial increase in data volume, posing significant challenges to computer systems in terms of data processing, storage, and retrieval. The bloom filter, a well-established probabilistic data structure, serves the purpose of determining the presence of a data element within a collection. This data structure offers notable advantages such as efficient space utilization and expedited query speed, rendering it extensively employed in various domains such as big data analysis [1], optimization of data storage in the Internet of Things [2], and safeguarding data security in cloud computing [3]. However, the conventional bloom filter exhibits a false positive probability during member queries, wherein an element that does not actually belong to the set is erroneously identified as a member of the set. As depicted in Figure 1, the bloom filter essentially comprises a binary vector *V* of a specific size *m* and multiple hash mapping functions *H*. The size of the bloom filter needs to be predetermined based on the number of data elements *E* to be stored and the desired probability of false positives, which poses challenges in accurately estimating the size of the stored elements in a system. The increase in false positives is observed in a bloom filter upon surpassing a specific threshold of stored elements. Fixed-size bloom filters are prone to significant storage space wastage or elevated false positives. Consequently, the expandability of bloom filters holds substantial research importance.

To address the aforementioned issues, previous studies have put forth over 60 enhanced bloom filters with the aim of enhancing their adaptability and performance [4]. These enhanced bloom filters exhibit enhanced flexibility and performance benefits in various application scenarios, including content caching [5,6,7,8,9,10], data storage [11,12,13,14,15], and other domains. By reducing false positives, optimizing implementation, augmenting collection diversity, and enriching functionalities, these enhanced bloom filters offer improved adaptability and efficacy, thereby rendering bloom filters a more potent tool for handling extensive datasets.

Despite the enhanced effectiveness of bloom filters in mitigating false positives, their ability to address computational overhead and additional space consumption remains limited. To tackle this issue, dynamic capacity expansion serves as a mechanism that facilitates automatic expansion of the bloom filter’s capacity in line with the growth of data volume. This approach not only significantly enhances the scalability of processing extensive datasets but also diminishes the rate of misjudgment. A robust dynamic expansion is crucial for enhancing the applicability of bloom filters and is a pivotal consideration in the development of extensive data processing systems.

The *p-adic* number serves as a valuable parameterization tool for computations within hyper-dimensional spaces [16]. Not only does it possess commendable algebraic properties, but it also forms a finite commutative ring with addition and multiplication operations while exhibiting favorable topological characteristics. The authors utilize the algebraic and topological characteristics of *p-adic* numbers to present a pioneering and scalable approach to bloom filters. The primary contributions are as follows:

1. The proposal of a bloom filter automatic expansion technique founded on *p-adic* integers. This technique enables the bloom filter to dynamically adapt its size in accordance with the actual number of stored elements, thereby resolving the problems associated with inefficient storage utilization and elevated false positive rates encountered in bloom filters employing fixed-size binary vectors;
2. Algorithms for the insertion and querying of a bloom filter, utilizing *p-adic* integers, have been developed, and their effectiveness in terms of storage and query efficiency has been validated through experimental analysis;
3. The algorithms address the limitation of conventional bloom filters in terms of expandability when the number of stored elements is uncertain, thereby enhancing the practicality and adaptability of bloom filters.

This paper is composed of five main sections. Section 1, Introduction, provides an in-depth exploration of the research purpose, existing issues, and the authors’ contributions in the field of bloom filters. Section 2, Related Works, critically analyzes the current research status and proposes research directions aimed at optimizing the performance of bloom filtering. Section 3, Methods, elaborates on the design ideas and algorithms employed in the *p*-adic Integer Scalable Bloom Filter (PSBF) in a comprehensive manner. Section 4, Results, presents the execution of the selection experiment conducted to determine the suitable hash function and the optimal prime *p* for the PSBF. Section 5, Discussion, presents a comparative and analytical examination of the performance tests conducted on PSBF and three other bloom filters. Additionally, Section 6, Conclusion, provides a summary of the advantages exhibited by PSBF.

## 2. Related Works

In contrast to conventional bloom filters, scalable bloom filters possess the ability to adjust their size dynamically in order to accommodate expanding data scales. Almeida et al. [17] addressed the issue of capacity limitations by proposing the utilization of multiple bloom filters to construct an expandable bloom filter. Nonetheless, this approach presents potential limitations, primarily including the division of data into multiple partitions and the requirement for query operations to be conducted across multiple partitions, thereby augmenting the query overhead. Additionally, the maintenance of multiple bloom filters necessitates additional storage overhead.

The dynamic bloom filter, as proposed by Guo et al. [18], is a resizable bloom filter that adjusts its size based on the cardinality of the designated dataset, resulting in a reduced rate of misjudgment. However, the dynamic resizing process of the bloom filter may incur additional time overhead due to memory allocation and data rearrangement. Furthermore, the inclusion of counters or bitmaps for maintaining the bloom filter introduces storage overhead. In response to these challenges, Patgir et al. [19] introduced rFilter, a member filter that offers excellent expandability and efficient utilization of space. In contrast to the conventional bloom filter, rFilter diminishes the likelihood of false positives; however, it is constrained in its capacity to retain bit-sharing information to only twice, thereby limiting its ability to share such information.

Kleyko et al. [20] conducted an analysis on the boundaries of false positives and true positives in the counting bloom filter and introduced a technique known as the Autoscaling Bloom Filter (ABF). The capacity of ABF can be adjusted based on the probability boundary of false positives and true positives, thereby enhancing the extensibility of conventional bloom filters by relaxing the need for a perfect true positive rate. ABF achieves this by sacrificing a non-zero true positivity rate in exchange for a reduced false positivity rate, which restricts the potential application scenarios where a non-zero true positivity rate is acceptable.

Kim et al. [21] employed the cyclic displacement method to enhance the operational speed of the standard bloom filter for both “add” and “query” operations while maintaining the same false positive rate. However, it should be noted that this method solely focuses on improving the computational efficiency of the standard bloom filter and does not address the persistently high false positive rate observed in the fixed-length bloom filter when processing extensive data. In contrast, Rottenstreich et al. [22] utilized Orthogonal Latin Square codes, and polynomials defined modulo primes to represent data elements, thereby leveraging the linear memory dependence of the dataset to accommodate a larger number of data elements. Despite its ability to prevent false positives in cases where the number of collection elements is below the set threshold, this method fails to account for the dynamic nature of the dataset. In response to this limitation, Nayak et al. [23] introduced a bloom filter known as RobustBF, which enhances the selection of hash functions and incorporates them into the 2D bloom filter [24] to minimize false positives and optimize memory usage. However, as RobustBF is currently implemented as a single-threaded system, its performance may require further enhancements to effectively handle high-throughput or low-latency data streams.

Dayan et al. [25] successfully incorporated InfiniFilter into the existing quotient filter [26]. This implementation utilizes a hash table to store the fingerprint of each entry, and its adaptable hash slot format ensures consistent functionality for “insert”, “query”, and “delete” operations. However, as the data volume increases, InfiniFilter’s performance in the “insert” operation falls short compared to the original quotient filter. Cohen et al. [27] introduced the concept of Spectral Bloom Filters (SBF), wherein the bit vector of the conventional bloom filter is extended to incorporate a counter vector. The functionality of querying and deleting SBF elements is facilitated by this counter operation. It is worth noting that both SBF and standard bloom filters are constrained by the dimensions of the bit vector. Kiss et al. [28] introduced the EGH filter, a data structure that enables bloom filter operations while ensuring error-free operations within a constrained set and a limited number of stored elements. However, the design limits the capability to handle dynamic collections.

These observations highlight the presence of challenging obstacles in the dynamic expansion mechanism of the bloom filter. The urgent need for comprehensive research lies in determining the optimal balance between the computing and memory overhead resulting from expansion, as well as devising strategies to minimize the occurrence of false positives in the filter.

## 3. Methods

This study proposes a solution to the issues associated with fixed-size bloom filters in previous works, namely the excessive waste of storage space and high false positive rates. Drawing inspiration from the design of the quotient filter [26], the authors introduce the PSBF. By increasing the prime number *p* and depth *d*, the PSBF dynamically expands to accommodate the storage elements, thereby optimizing the utilization of storage space.

**Figure 2:**
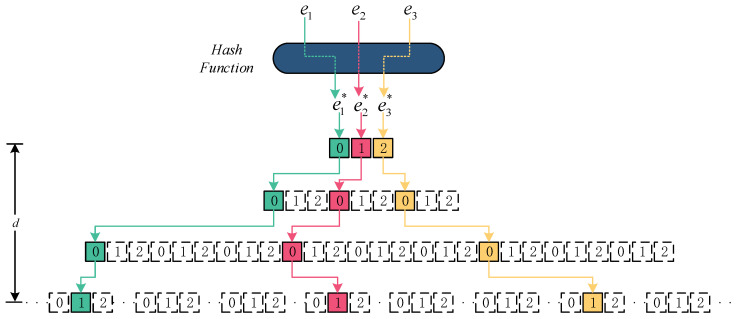
Data structure of *3-adic* Integer Scalable Bloom Filter.

As depicted in Figure 2, the data structure of the PSBF primarily consists of a tree logical structure comprising a linked list and three key operations: “initialize”; “insert”; and “query”. Initially, a Hash Function is employed to map any data element e to an integer $e^{*}$. Subsequently, this integer is transformed into a *p-adic* integer using a prime number *p*. Furthermore, the depth *d* of the bloom filter is determined based on the number of bits present in the converted *p-adic* integer. Consequently, the storage structure of the PSBF adopts a tree structure, thereby circumventing the linear storage structure employed in previous methodologies. This novel approach facilitates more efficient element management.

1. The *p-adic* integer algebraic representation is defined as follows: Let *α* be an element in the number domain ***Q****_p_*. It can be expressed as Equation (1).

(1) $$\alpha =\sum_{i=n}^{\infty }a_{i}p^{i}$$

where *p* is a prime number, *n* is an integer, and *a_i_* ∈ **Z**. If *i* = 0 and *a_i_* ∈ {0,1,…,*p*−1}, then *α* is an integer, and there exists a unique *p-adic* decomposition for *α* in the case of modulo *p*. The *p-adic* expanded power polynomial is depicted in Equation (2).

(2) $$\alpha =a_{n}p^{n}+a_{n+1}p^{n+1}+a_{n+2}p^{n+2}+\ldots$$

It is utilized to represent *α*. Specifically, the *p-adic* integer is chosen in the PSBF, with *n* set to 0. Consequently, each coefficient is proposed to form a representation of its corresponding coefficient, as illustrated in Equation (3).

(3) $$\alpha =a_{n}a_{n+1}\ldots a_{n-2}a_{n-1}.a_{0}a_{1}a_{2}\ldots$$

where the point between the coefficients *a_n−1_* and *a_0_* is referred to as the *p-adic* point, which exclusively represents the symbol for the *n* value, while *a_i_* is denoted as the *p-adic* number.

2. Coefficient extraction operation. Taking the example of *3-adic*, the *p-adic* integer representation is obtained. Let an integer *e* = 11 be given, and the *3-adic* expanded form of the number *e* is calculated using Equation (2), where *n* = 0. The process involves three steps:

step 1 *α*≡*a*_0_(mod 3^0^) → *a*_0_ = 2;

step 2 *α*≡*a*_1_(mod 3^1^) → *a*_1_ = 0;

and step 3 *α*≡*a*_2_(mod 3^2^) → a_2_ = 1.

After completing these steps, the result is 11 = 2×3^0^ + 0×3^1^ + 1×3^2^, yielding the *3-adic* number *α* as 0.201.

3. Calculation of the probability of false positives. The PSBF involves determining the depth *d* and prime number *p* of the *p-adic* expansion. The number of slices from layer 1 to layer *d* is represented by *p*, *p*^2^, *p*^3^, …, *p^d^*. The probability of a false positive for a single number *P_f_* is calculated using Equation (4).

(4) $$P_{f}=\prod_{i=1}^{d}\frac{1}{p^{i}}$$

while the probability that the number does not have a false positive *P_t_* is denoted by Equation (5).

(5) $$\;P_{t}=1-\prod_{i=1}^{d}\frac{1}{p^{i}}$$

Furthermore, the probability that *n* numbers do not have false positives $P_{t}^{n}$ is denoted by Equation (6).

(6) $$P_{t}^{n}={\left(1-\prod_{i=1}^{d}\frac{1}{p^{i}}\right)}^{n}$$

The calculation process for determining the probability *P* of false positives for *n* numbers is illustrated in Equation (7).

(7) $$P=1-{\left(1-\prod_{i=1}^{d}\frac{1}{p^{i}}\right)}^{n}$$

Hence, the likelihood of encountering false positives in a bloom filter is inversely related to the magnitude of the expanded prime number *p* and depth *d* while being directly proportional to the number *n* of stored elements.

4. PSBF operation. During the initialization stage, the algorithm “InitBloom” is executed to generate a *bt* bloom filter. As depicted in Algorithm 1, a “*tree*” array is initialized. Each data element comprises two attributes, namely “*Id*” and “*Index*”, where “*Id*” represents the index and *“Index”* denotes the array of its own structure.

**Algorithm 1** InitBloom**Input:** none**Output**: *bt*1.**function** InitBloom2.  *bt*⇐create an empty array *tree*;3.  **return**
*bt*4.**end function**

In the stage of inserting data elements, an element must first undergo mapping using a string hash function. The hash function employed in this study is BKDRHash [29], which is capable of transforming any string into an integer. The algorithm used for this conversion is Algorithm 2.
**Algorithm 2** BKDRHash**Input:** *str***Output**: *hashCode*1.**function** BKDRHash (*str*)2.  *seed*⇐131;3.  *hash*⇐0;4.  **for**
*i*⇐0 **to** len(*str*) - 1 **do**5.    *hash*⇐(*hash*×*seed*) + unit64(*str*[*i*]);6.  **end for**7.  **return**
*hashCode*&*0x7FFFFFFF*8.**end function**

Algorithm 3 delineates the sequential procedures entailed in the conversion of integers to *p-adic* integers. The process commences with the establishment of an empty string “*new_num_str*”, designated for the storage of the converted outcome. Furthermore, the variables “reminder” and “*reminder_string*” are introduced to accommodate the retention of the residual value of the present bit and its corresponding string representation, correspondingly. Subsequently, the loop should be executed until the input integer “*num*” reaches 0, signifying the termination of the loop. Within each iteration, the remainder of the expanded prime number *p* divided by “*num*” should be obtained to determine the current bit’s remainder. It is imperative to ascertain whether the remainder falls within the range of 10 to 75. If it does, the corresponding character representation in the predefined mapping table “*tenToAny*” should be identified. Conversely, if the remainder does not fall within this range, it should be converted into a string representation. The resulting current bit string, “*remainder_string*”, should then be appended to the end of “*new_num_str*”. The prime number *n* is used to divide “*num*”, resulting in an updated value of “*num*”. Upon completion of the loop, the converted string representation of a *p-adic* integer is stored in “*new_num_str*” and returned.
**Algorithm 3** DecimalToAny**Input:** *num*, *p***Output**: *new_num_str*1.**function** DecimalToAny(*num*,*p*)2.  initialize variables *new_num_str*, *remainder*, *remainder_string*;3.  **while**
*num*≠0 **do**4.    *remainder*⇐
*num* mod *p*;5.    **if**
*remainder* > 9 **and**
*remainder* < 76 **then**6.      *remainder_string*⇐*tenToAny*[*remainder*];7.    **else**8.      *remainder_string*⇐str(*remainder*);9.    **end if**10.    *new_num_str*⇐
*tetany + remainder_string*;11.    *num*⇐*num*/*p*;12.   **end while**13.   **return**
*new_num_str*14. **end function**

**Figure 3:**
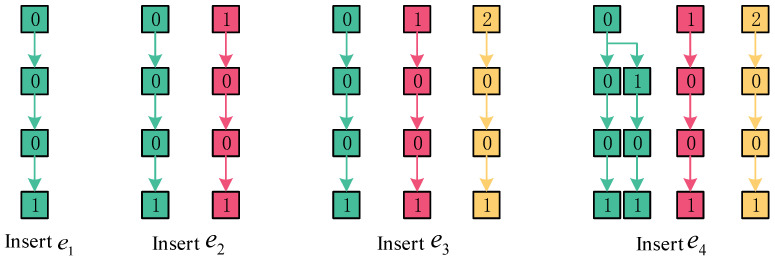
Storage status of the PSBF.

PSBF element inserted. Let elements $e_{1}$ = 27, $e_{2}$ = 28, $e_{3}$ = 29, and $e_{4}$ = 30, then Algorithm 3 is executed to obtain their respective *3-adic* integers: 0.0001; 0.1001; 0.2001; and 0.0101. The specific steps for inserting these elements into the bloom filter are illustrated in Figure 3.

The initial digit of the *p-adic* integer of the element $e_{1}$ is determined to be 0. In the event that no slice with an index of 0 is present in the first layer, a new slice will be appended and assigned an index of 0. Similarly, the second digit is also determined to be 0. If no slice with an index of 0 is found in the second layer, a new slice will be added, and the corresponding index will be assigned as 0. The third digit is likewise determined to be 0. In the event that no slice with an index of 0 is found in the third layer, a new slice will be appended and assigned an index of 0. Finally, the fourth digit is determined to be 1. If a slice with an index of 1 is not located in the fourth layer, a new slice will be appended, and the corresponding index will be assigned a value of 1. The verification process confirms the input of all bits, thereby completing the storage of the element. Similarly, the storage of elements $e_{2}$ and $e_{3}$ can be accomplished. For the *p-adic* integer of the element$e_{4}$, the initial position is 0. In the first layer, a slice with an index of 0 is identified, prompting a transition to the subsequent layer of the slice. In the second position, where the index is 1, no slice is present in the second layer of the slice. Consequently, a new slice will be appended, and the corresponding index will be assigned as 1. Similarly, in the third position, where the index is 0, no slice is detected in the third layer. As a result, a new slice will be appended, and the corresponding index will be assigned as 0. In the fourth position, where the index is 1, no slice is found in the fourth layer. Therefore, a new slice will be appended, and the corresponding index will be assigned as 1. Verify and ensure that all bits have been inputted, and finalize the *p-adic* integer storage of the element$e_{4}$. The precise methodology is illustrated in Algorithm 4.
**Algorithm 4** InsertElement**Input:** *e, bt***Output**: *newBt*1.**function** InsterElement(*e*, *bt*)2.  initialize variables *path*, *flag*, *num*, *newBt*;3.  **for**
*i*⇐0 **to** len(*e*)—1 **do**4.    **for**
*h*⇐0 to len(*bt*))—1 **do**5.      **if** string(*e*[*i*]) == *bt*[*h*].*Id* **then**6.        *flag*⇐true; 7.        **if** *i* =- 0 **then**8.          *path*⇐**strconv.Itoa**(*h*) + “*.Index*”9.        **else**10.          *path*⇐*path* + “.”+ **strconv.Itoa**(*h*) + “*.Index*”;11.        **end if**12.        *bt*⇐*bt*[*h*].*Index;*13.        **break**14.      **else**15.        *flag*⇐false;16.      **end if**17.    **end for**18.    **if** !*flag* **then**19.      **if** *i* == 0 **then**20.       create *a* new node *a* of the tree, add a to the end of *newBt*, record *path*, and *bt*⇐*newBt* [*len(newBt)-1*].*Index;*21.       **else**22.       create a new node *a* of the tree, add *a* to the end of *bt*, update *newBt*, and *bt*⇐*bt* [*len(bt)-1*].*Index;*23.         **if**
*num* == 0 **then**24.          *path*⇐*path* + “.”+ **strconv.Itoa**(*v*) + “*.Index*”;25.          *num*++;26.         **else**27.          *path*⇐ *path* + “.0*.Index*”;28.         **end if**29.        **end if**30.       **end if**31.   **end for**32.   **return** *s*33.**end function**

The “SearchElement” algorithm, which is a component of the element query algorithm, bears a resemblance to the “InsertElement” algorithm. It employs the same hash function utilized in the stored procedure to convert the queried element into an integer. The selection of the expanded prime number *p* remains consistent with the one used during the element storage process, and the integer resulting from the element mapping is expanded to derive the *p-adic* integer of the element. The detailed implementation of this process is presented in Algorithm 5.
**Algorithm 5** SearchElement**Input:** *e, bt***Output:** true or false1.**function** SearchElement(*e*, *bt*)2.  for i⇐0 **to** len(*e*)—1 **do**3.    **if** len(*bt*) < 1 **then**4.      **return** false5.    **end if**6.    *found*⇐ false;7.    **for**
*h*⇐0 **to** len(*bt*)—1 **do**8.      **if** string(*e*[*i*]) = *bt*[*h*].*Id* **then**9.         *found*⇐true;10.       *bt*⇐ *bt*[*h*].*Index;*11.       **break**12.      **end if**13.    **end for**14.    **if** !*found* **then**15.      **return** false16.    **end if**17.   **end for**18.   **return** true19.**end function**

## 4. Results

All experiments conducted in this study were performed within a consistent environment, utilizing the following main hardware configuration: Intel(R) Core(TM) i5-8250 U CPU @ 1.60 GHz, 1.80 GHz, and 12 GB RAM. The operating system employed wazs Windows 11 64-bit, and the programming language utilized was Golang 1.18.4. In order to achieve optimal performance of the PSBF, various prime numbers *p* and hash functions were chosen for experimentation in this study.

### 4.1. Selection of the Optimal Prime Number p

**Figure 4:**
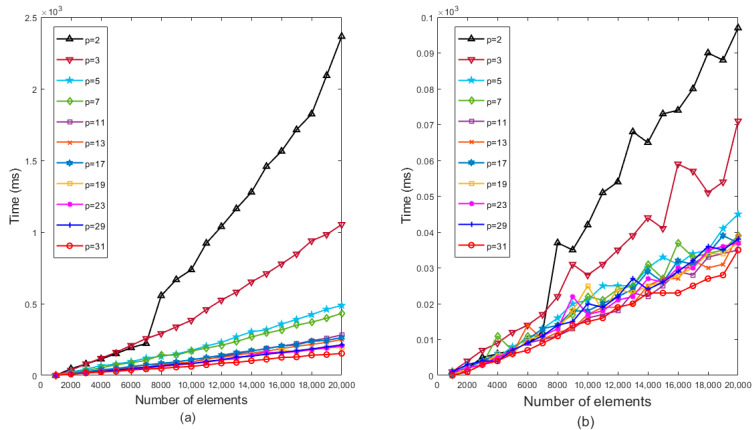
Time comparison of selecting different expansion prime numbers *p* from 2 to 31 intervals. (**a**) Insertion operation; (**b**) query operation.

For this study, the hash function’s output result length was set at 32, thus limiting the prime number p within the range of 2 to 31. The experiment employed a comparative analysis of insertion and query operations to determine the minimum prime number *p* that yields the optimal performance in a PSBF. Figure 4a,b demonstrates the impact of selecting prime number *p* on the insertion and query capabilities of the bloom filter when inserting 1000 to 20,000 data elements. As the number of elements increases, both insertion and query times gradually rise, albeit with slight variations in the performance observed across different prime numbers p. In the context of insertion and query operations, the prime number *p* with a value of 31 demonstrated optimal performance and exhibited a brief execution time. Conversely, prime numbers such as 2, 3, 5, 7, etc., tended to exhibit a prolonged execution time, particularly when the number of elements was substantial, thereby resulting in a more pronounced degradation in performance. Consequently, this study advocates for the utilization of the expanded prime number *p* = 31 within the bloom filter as it yields superior performance and efficiency.

### 4.2. Selection of a Suitable Hash Function

The determination of the most suitable number of hash functions for conventional bloom filters is contingent upon the quantity of inserted elements and the dimensions of the bit array employed by said filters [30]. In contrast, the hash function employed by the PSBF diverges from that of traditional bloom filters. Specifically, the hash function in the PSBF transforms the elements to be manipulated into integers of fixed length. As a result of the incorporation of *p-adic* numbers in the PSBF, it becomes solely necessary to decompose the fixed-length integers into *p-adic* numbers based on the optimal prime number *p*. The PSBF does not internally utilize a hash function. In essence, the utilization of the PSBF does not necessitate the consideration of the number of elements or the dimensions of the bit array within the bloom filter. The selection of a suitable hash function for the PSBF pertains to the efficiency of operations performed on elements that are mapped to fixed-length sequences of integers.

**Figure 5:**
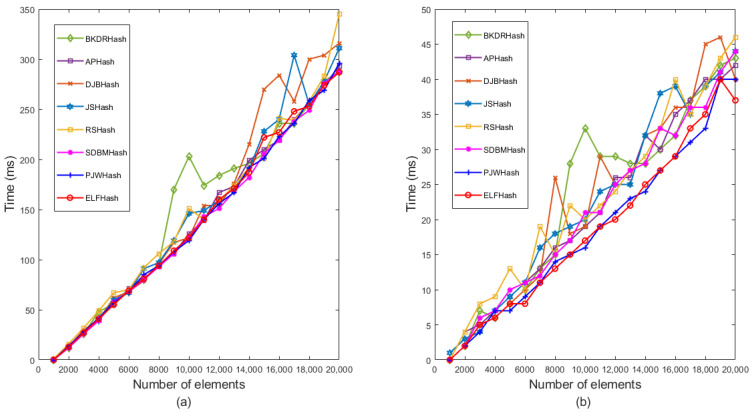
Time comparison of eight various hash functions. (**a**) Insertion operation; (**b**) query operation.

The experiment involved the insertion and query of 1000–20,000 identical data elements using eight string hash functions, namely BKDRHash [29], APHash [31], DJBHash [32], JSHash [33], RSHash [34], SDBMHash [35], PJWHash [36], and ELFHash [37]. The results of these experiments are illustrated in Figure 5a and Figure 5b, respectively. BKDRHash, APHash, JSHash, SDBMHash, and PJWHash exhibited favorable performance in terms of insertion and query time, displaying relatively consistent performance. However, DJBHash was characterized by a prolonged insertion and query time, rendering it unsuitable for large-scale data insertion and query operations in the PSBF. RSHash demonstrated an even higher query time, with a significant decline in performance when dealing with substantial data volumes. Although ELFHash exhibited relatively high insertion and query times, its growth rate was comparatively gradual.

## 5. Discussion

It is worth noting that the conventional bloom filter exhibits a false positive probability during member queries, wherein an element that is not present in the set is erroneously identified as being part of the set. Furthermore, the size of the bloom filter necessitates pre-definition based on the desired probability of false positives and the number of elements to be stored. Estimating the accurate size of the stored elements in a system poses a challenge. When the number of elements stored in a bloom filter exceeds a specific threshold, the occurrence of false positives will escalate. The utilization of fixed-size bloom filters will inevitably result in significant storage wastage or a high rate of false positives. To ascertain the superiority of the PSBF, this study undertakes a comparative analysis of insertion time, query time, space utilization rate, and misjudgment count in relation to standard bloom filters, scalable bloom filters [17], and dynamic bloom filters [18].

### 5.1. Analysis of Insertion and Query Performance

**Figure 6:**
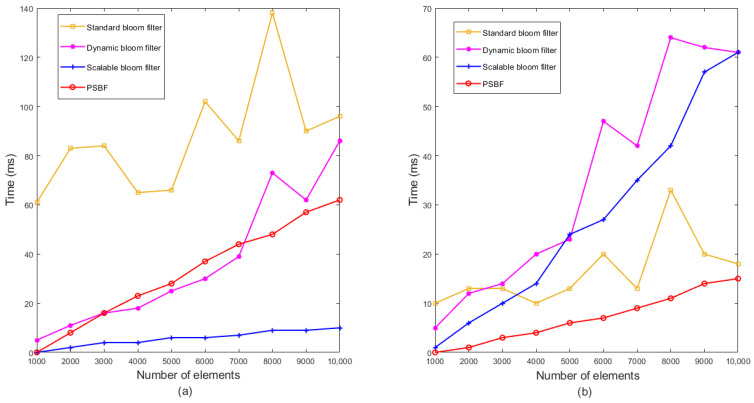
Time comparison of the different bloom filters. (**a**) Insertion operation; (**b**) query operation.

Regarding the insertion operation, as depicted in Figure 6a, the insertion time of the four distinct filters exhibited a gradual increase as the number of elements grew. Nonetheless, this increase remained relatively low, with the time being consistently below 0.14 ms. As the data elements increased, both the PSBF and expandable bloom filters demonstrated more consistent performance, while standard bloom filters and dynamic bloom filters exhibited comparatively higher insertion times. Furthermore, the insertion performance of the system exhibited significant fluctuations with an increase in the number of inserted elements. Regarding query operations, as depicted in Figure 6b, the query time for the four bloom filters gradually increased as the number of elements grew. Dynamic bloom filters demonstrated relatively high query times, while the PSBF and scalable bloom filters exhibited relatively stable query performance. However, the PSBF outperformed the other three bloom filters in terms of both query performance and time.

### 5.2. Analysis of Space Utilization and Misjudgment Number

**Figure 7:**
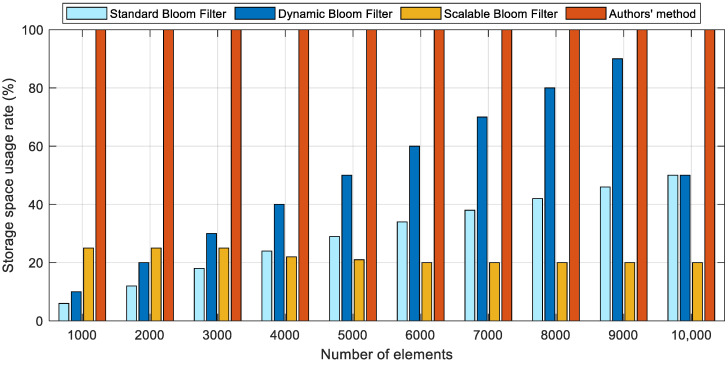
Storage space usage rate of four various bloom filters.

In relation to space utilization, as depicted in Figure 7, the space utilization of both the standard bloom filter and the dynamic bloom filter progressively rose with the augmentation of inserted elements. Nevertheless, upon reaching the predetermined value of inserted elements, the dynamic bloom filter, in order to maintain a false positive probability lower than the anticipated threshold, generated a bloom filter with greater space and allocated a substantial amount of free space, consequently leading to a sudden decrease in space utilization. The space utilization rate of the expandable bloom filter will exhibit a gradual decline as the number of inserted elements increases. Once the space utilization rate surpasses a specific threshold, a larger bloom filter will be generated and stored at the forefront of the bloom filter set, serving as an active bloom filter [4]. Consequently, this leads to increased available space for expandable bloom filters and a reduction in space utilization.

This study employed the PSBF method to initially convert the target element into an integer, followed by the selection of an expanded prime number *p* to convert the integer into a p-adic integer further. The depth *d* of the resulting bloom filter was determined based on the number of bits in the converted *p-adic* integer. The storage structure of the PSBF was transformed into a tree, deviating from the linear storage structure employed in current technologies. This modification enhances the efficiency of element insertion and query operations. Furthermore, the PSBF employs a selection process to determine the expanded prime number *p* based on the given integer, which is then utilized to convert the integer into a *p-adic* integer. Subsequently, the depth *d* of the bloom filter is determined based on the number of bits in the converted *p-adic* integer. This approach allows the PSBF to circumvent the need to consider the size of the bloom filter and the stored data, enabling automatic expansion of capacity and storage of any number of elements based on the storage elements. Consequently, the PSBF effectively utilizes the available storage space.

**Figure 8:**
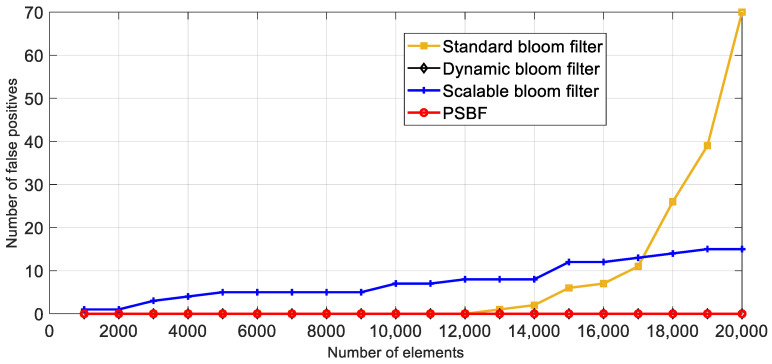
Number of misjudgments of four different bloom filters.

Based on the findings presented in Figure 8, it is evident that the standard bloom filter lacks the capability of automatic expansion. Consequently, when the space utilization rate is high, the likelihood of false positives in the bloom filter increases significantly. Therefore, in order to maintain a lower-than-anticipated probability of false positives, the standard bloom filter must compromise its space utilization. The dynamic bloom filter consistently recorded zero misjudgments, whereas the expandable bloom filter exhibited a gradual linear increase in misjudgments. The PSBF algorithm selected an appropriate expanded prime number *p* by considering the integer obtained from the hash function, thereby minimizing false positives. When the *p-adic* integer depth of all data conversions was equal, it became possible to attain a probability of zero false positives.

As previously stated, the PSBF demonstrates clear advantages in terms of query time, space utilization, and number of misjudgments. It consistently exhibited shorter query times, better space utilization, and fewer misjudgments when compared to the scalable bloom filter, regardless of the size of the element set or the number of elements. Although the insertion operation time of the PSBF was slightly higher than that of the scalable bloom filter, in practical application scenarios, the insertion operation time of the bloom filter was typically significantly smaller than the query operation time. Consequently, the overall performance of the PSBF surpasses that of the scalable bloom filter.

## 6. Conclusions

To address the limitations of capacity in the existing bloom filter, it becomes unfeasible to reprocess extensive data for filtration. Moreover, when the data to be filtered approaches the upper limit of capacity during the reprocessing process, the false positive rate significantly rises, leading to a substantial decline in the efficacy of data filtration. Consequently, this gives rise to technical challenges that diminish the accuracy of the filtering process. This study introduced a bloom filter with an automatic expansion tree structure, utilizing the algebraic and topological properties of *p-adic* integers. The proposed approach offers improved efficiency in terms of element insertion and query operations. Moreover, it eliminates the need for parameter setting to accommodate capacity expansion, thereby mitigating space wastage and reducing the likelihood of false positives.

## Data Availability

The data from this study can be obtained upon request from the first or corresponding author.
